# Ubenimex enhances Brd4 inhibition by suppressing HEXIM1 autophagic degradation and suppressing the Akt pathway in glioma cells

**DOI:** 10.18632/oncotarget.17314

**Published:** 2017-04-21

**Authors:** Liping Han, Qingwei Zhao, Xianhong Liang, Xiaoqing Wang, Zhen Zhang, Zhiguo Ma, Miaoqing Zhao, Aihua Wang, Shuai Liu

**Affiliations:** ^1^ Department of Urology, Shandong Provincial Hospital Affiliated to Shandong University, Jinan, Shandong, P.R. China; ^2^ Department of Neurology, Qianfoshan Hospital Affiliated to Shandong University, Jinan, Shandong, P.R. China; ^3^ Department of Neurology, Shandong Police Hospital, Jinan, Shandong, P.R. China; ^4^ Department of Pediatric Surgery, Shandong Provincial Hospital Affiliated to Shandong University, Jinan, Shandong, P.R. China; ^5^ Department of Neurosurgery, Shandong Provincial Hospital Affiliated to Shandong University, Jinan, Shandong, P.R. China; ^6^ Department of Pathology, Shandong Provincial Hospital Affiliated to Shandong University, Jinan, Shandong, P.R China

**Keywords:** glioma, BRD4 inhibition, autophagy, HEXIM1, Akt pathway

## Abstract

Inhibition of Brd4 by JQ1 treatment showed potential in the treatment of glioma, however, some cases showed low sensitivity of JQ1. In addition, the pre-clinical analysis showed its limitation by demonstrating that transient treatment with JQ1 leads to aggressive tumor development. Thus, an improved understanding of the mechanisms underlying JQ1 is urgently required to design strategies to improve its efficiency, as well as overcome its limitation. HEXIM1 has been confirmed to have an important role in regulating JQ1 sensitivity. In our study, ubenimex, a classical anti-cancer drug showed potential in regulating the JQ1 sensitivity of glioma cells using the WST-1 proliferation assay. Further studies demonstrated that ubenimex inhibited autophagy and downregulated the autophagic degradation of HEXIM1. The role of HEXIM1 in regulating JQ1 sensitivity was verified by the HEXIM1 knockdown. Since ubenimex was verified as an Akt inhibitor, we further studied the role of Akt inhibition in regulating JQ1 sensitivity and migration of glioma cells. Data showed that ubenimex improved the efficiency of JQ1 treatment and suppressed migration both in the *in vitro* and *in vivo* xenografts models. The Akt agonist attenuated these effects, pointing to the role of Akt inhibition in JQ1 sensitivity and suppressed migration. Our findings suggest the potential of ubenimex adjuvant treatment to enhance JQ1 efficiency and attenuate parts of its side effect (enhancing tumor aggressive) by regulating the autophagic degradation of HEXIM1 and Akt inhibition.

## INTRODUCTION

Gliomas represent about 30% of brain and central nervous system tumors. While the rate of successful treatment of gliomas is lower, most often, high-grade tumors show dismal prognosis. For the past 20 years, there has been little progress in clinical trials testing new agents until a novel Brd4 inhibition by JQ1 treatment showed potential in glioma treatment [1, 2]. However, some cases showed a low sensitivity of JQ1 [3, 4], while pre-clinical analysis showed its limitation by demonstrating that transient treatment with JQ1 leads to aggressive tumor development and, consequently, accelerated death [5].

BRD4 belongs to the BET (bromodomain and extraterminal domain) subfamily of bromodomain-containing proteins comprised of Brd2, Brd3, Brd4, and Brd5.BRD4 protein is elevated in gliomas and has been demonstrated to regulate transcriptional activation of various genes involved in sustaining growth and developmental of tumors, and therefore, drugs targeting BET show potential therapeutic intervention. Brd4 functions by recruiting the p-TEFb protein complex, which is composed of cyclin-dependent kinase 9 and its regulatory partner cyclin T1. P-TEFb protein complex is essential for the activation of promoters and super-enhancers by interacting with acetylated histones [6–8]. Multiple evidences suggest that Brd4 regulates transcription elongation carried out by RNA Polymerase II as well as expression of oncogenes including Bcl2 and c-Myc [8–10]. HEXIM1(hexamethylene bisacetamide-inducible protein 1) negatively regulates the functional activity of p-TEFb [11, 12]. Homeostasis between the activation of Brd4 by p-TEFb and inhibition of p-TEFb by HEXIM1 determines the extent of BET activation [8, 9, 11, 12]. Interestingly, recent work suggests that activation of PI3K/AKT pathway can inhibit interaction of HEKIM1 with P-TEFb, and therefore, enhance Brd4 activation [13]. Besides, the latest research has demonstrated that HEXIM1 are degraded through the process of autophagy in a number of acute leukemia cell lines [14]. In turn, inhibition of autophagy substantially increases the expression of HEXIM1, suggesting that the activation of autophagy confer sensitivity to BET inhibitors.

Ubenimex (Bestatin) is a low-molecular-weight dipeptide that enhances the function of immunocompetent cells and exhibits diverse effects on the production of cytokines. It shows benefits in glioma treatment by inhibiting the 5-LO-LTA4 hydrolase pathway [15]. Our previous study demonstrated its function as an Akt inhibitor and a regulator of autophagy in RCC and prostate cancer cells [16–18]. In this study, we have discovered that ubenimex suppresses the Akt pathway and autophagy in glioma cell lines, resulting in reduced proteolysis of HEXIM1, increasing HEXIM1 expression and activity. Our results suggest that regulation of the Akt pathway and autophagy in glioma cells confers sensitivity to BET inhibitors and provides a synergistic effect of JQ1 on glioma.

## RESULTS

### Ubenimex enhances the sensitivity of JQ1 (BRD4 inhibition)-induced cell death by upregulating HEXIM1 expression in glioma cells

#### Ubenimex enhances the sensitivity of JQ1-induced growth inhibition and apoptosis

**Figure 1:**
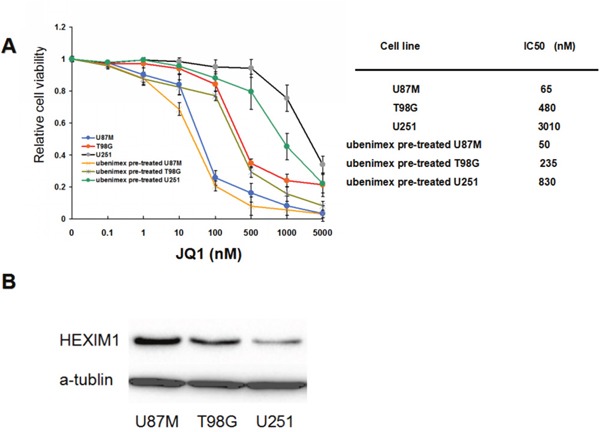
**(A)** U87M, T98G, and U251, were selected for Western blot. U87M cells showed a high level of HEXIM1 expression, while U251 showed the lowest expression of HEXIM1. **(B)** WST-1 cell proliferation. All 3 glioma cell lines were pre-treated by ubenimex (0.5mg/ml, 48 hours) before the test. Cells were treated with JQ1 at varying concentrations (from 10nM to 5uM) for 72 hours then were subjected to WST-1 assay. IC50 was showed on the right.

To investigate the effects of JQ1 on glioma cell proliferation, we first employed the WST-1 assay to determine the effects of JQ1on glioma cell lines. Three cell lines of interest were subject to the test (U87M, T98G and U251). The cells were treated with JQ1 at varying concentrations (from 10nM to 5μM) for 72 hours. The WST-1 assay demonstrated a dose-dependent decrease in the viability of all three cell lines following treatment with JQ1 (P<0.05, Figure 1B). The IC50 values were 65 nM for U87M, 480 nM for T98G, and 3010 nM for U251. After 72 hours of JQ1 treatment, U87M showed the most sensitivity to JQ1, while U251 showed the least response to JQ1 treatment.

To investigate the effects of ubenimex on JQ1 sensitivity, we pre-treated the cells with ubenimex (0.5mg/ml) for 48 hours before treatment with JQ1. The WST-1 assay showed no cytotoxicity due to ubenimex pre-treatment alone on these three cell lines (data not show); however, the data showed an increase in JQ1 sensitivity (P<0.05, Figure 1A). The IC50 values shifted to 50 nM for U87M, 235 nM forT98G and 830 nM for U251.

#### HEXIM1 plays an important role in basal and ubenimex enhanced JQ1 sensitivity

Our study showed distinct sensitivity to JQ1 in three different glioma cell lines. As some reports showed, HEXIM1 expression and activity contribute to BRD4 inhibition; therefore, we tested HEXIM1 expression in these three types of cells by Western Blot. WB showed that U87M exhibited the highest HEXIM1 expression, while U251 exhibited the lowest expression (Figure 1B). This indicated that HEXIM1 plays an important role in basal JQ1 sensitivity.

**Figure 2:**
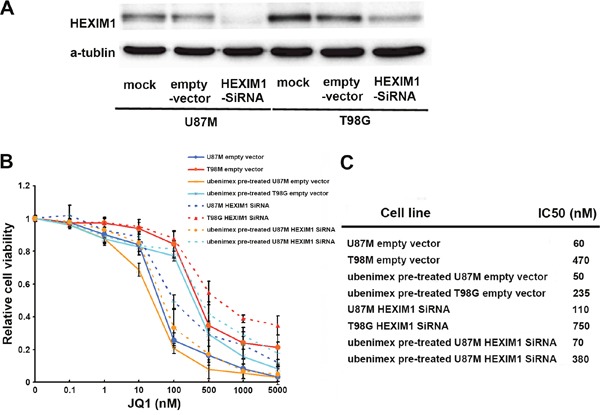
**(A)** Western Blot was carried out to test HEXIM1 knock down in U87M and T98G glioma cell lines. **(B)** WST-1 cell proliferation assay was used to test the effect of HEXIM1 on JQ1 sensitivity. **(C)** IC50 of JQ1 on all 3 groups.

To verify this concept, we down-regulated HEXIM1 expression using HEXIM1 SiRNA in U87M and T98G (basal HEXIM1 expression in U251 is very low). After verification using Western blot (Figure 2A), HEXIM1 downregulated cells (Si-HEXIM1), empty vectors (Si-control), and control cells were subjected to the WST-1 cell proliferation assay. There is no significant difference between the control (no treatment) and mock (empty vector transfected) groups. However, compared to the control group, both of these cell lines showed less JQ1 sensitivity after knock down of HEXIM1, (P<0.05, Figure 2B). The IC50 values shifted to 110 nM for U87M HEXIM1 SiRNA, and 750nM for T98G HEXIM1 SiRNA (Figure 2C). These results indicated that HEXIM1 plays an important role in JQ1 sensitivity.

In addition, we tested the ubenimex pre-treated cells in the same way to identify whether the HEXIM1 knockdown has the same effect on ubenimex-enhanced JQ1 sensitivity. Generally, all three groups (HEXIM1 SiRNA, empty vector and control cells) were pre-treated with ubenimex (0.5mg/ml, 48 hours), followed by JQ1 treatment at varying dose (10nM-5uM) for 72 hours. WST-1 assay showed that downregulating HEXIM1 could attenuate ubenimex-enhanced JQ1 sensitivity (P<0.05, Figure 2B). In comparison to ubenimex pre-treated cells, the IC50 for JQ1HEXIM1 knockdown groups ‘shifted to 70 nM for U87M HEXIM1 SiRNA, and 380 nM for T98G HEXIM1 SiRNA (Figure 2C).

**Figure 3:**
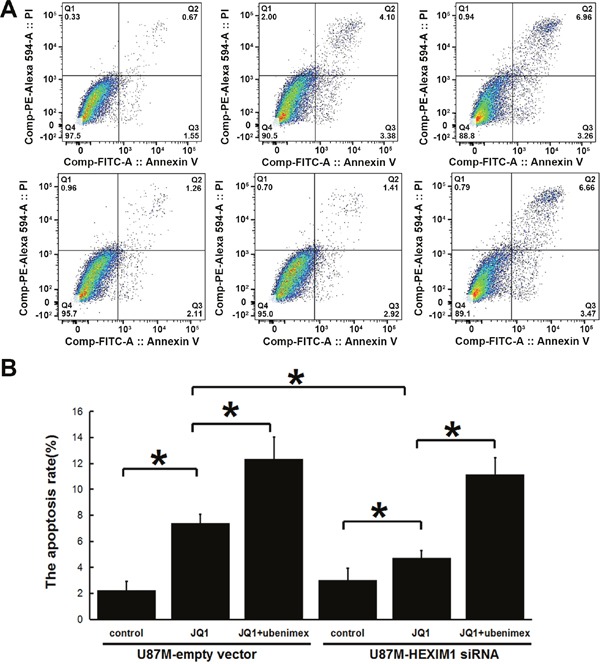
Annexin V-PI staining assessing U87M mock control and U87M HEXIM1 knockdown cells’ apoptosis rates after JQ1 or JQ1 combined ubenimex treatment JQ1 induced apoptosis in U87M cells, the effect attenuated by the downregulation of HEXIM1, indicating the role of HEXIM1 on JQ1 sensitivity. Ubenimex enhanced both mock control and HEXIM1 knockdown cells' JQ1 sensitivity. **(A)** Flow-cytograms; **(B)** quantitation of a (*P < 0.05).

**Figure 4:**
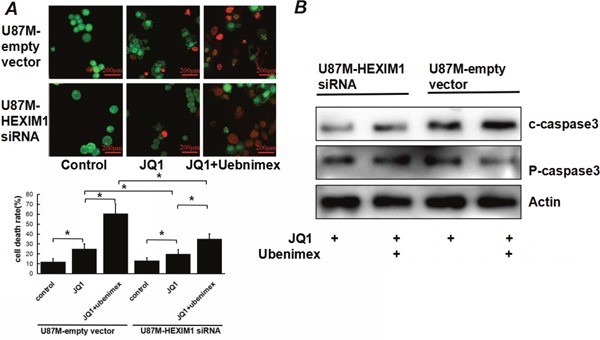
JQ1 induced apoptosis in U87M cells, the effect was attenuated by knocking down HEXIM1 **(A)** U87M (mock and HEXIM1 knock down) cells were treated with JQ1 alone or combine with ubenimex at a dose of 0.5 mg/ml for 16 h. We used fluorescence microscopy to examine DNA damage and cell death. The dead cells (red) per field determined by acridine orange staining are shown. **(B)** Western Bolt of caspase3 (c-capase3:cleaved caspase3; P-caspase3:pro-caspase3)showed the same trend of JQ1 or JQ1+unbenimex induced apoptosis.* P<0.05. Data are expressed as the mean ± standard deviation of 3 independent experiments.

Annexin V-PI staining assessing U87M mock control and U87M HEXIM1 knockdown cells' apoptosis rates after JQ1 or JQ1 combined ubenimex treatment. JQ1 induced apoptosis in U87M cells, the effect was attenuated by knocking down HEXIM1 which indicated a role of HEXIM1 on JQ1-induced apoptosis. Ubenimex enhanced the JQ1 sensitivity of both mock control and HEXIM1 knockdown cells (Figure 3). Acridine orange (AO)/ethidium bromide (EB) double staining shows the level of cell death, western blot of caspase3(including cleaved caspase3 and pro-caspase3) showed the same trend (Figure 4), we can find out that ubenimex can increase apoptosis and these effects can be attenuated by HEXIM1 knockdown.

#### Upregulation of HEXIM1 expression after ubenimex treatment by suppression of autophagic protein degradation

**Figure 5:**
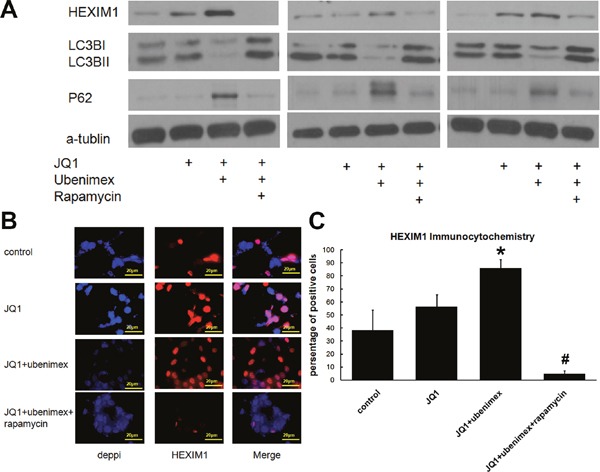
**(A)** Western Blot showed JQ1 or JQ1 combined ubenimex treatment on HEXIM1 and autophagy marker LC3B, P62 expression. Pharmacologic autophagy stimulator Rapamycin (10nm) attenuated this effect on degradation of HEXIM1. **(B)** Immunofluorescence staining of HEXIM1 on JQ1 or JQ1 combined ubenimex treatment on U87M cells. **(C)** Quantitation of B. *: P<0.05 vs control. #, P<0.05 vs JQ1+ubenimex.

We tested the effect of ubenimex on HEXIM1 expression using Western blot; the data pointed to significant HEXIM1 up-regulation after ubenimex pre-treatment in all three cell lines (Figure 5A).

**Figure 6:**
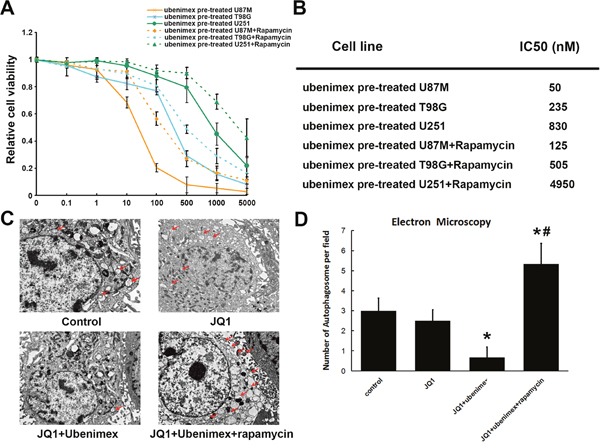
**(A)** WST-1 cell proliferation assay were used to test the effect of autophagy on JQ1 sensitivity. **(B)** IC50 of JQ1 showed autophagy stimulated by rapamycin attenuated ubenimex enhanced JQ1 sensitivity. **(C, D)** Number of autophagosome were observed by using electron microscopy. Data showed ubenimex significantly inhibited autophagy of U87M glioma cells.*: P<0.05 vs control. #: P<0.05 vs JQ1+ubenimex.

The up-regulation of HEXIM1 by ubenimex treatment, in conjunction with markedly increased expression of p62 and decreased LC3B expression, pointed to the downregulation of autophagy, a process through which proteins are targeted for degradation in autophagocytic vesicles (Figure 5A). Besides, a reduced count of autophagosomes was observed in the ubenimex-treated group using the electron microscope (P<0.01, Figure 6C, 6D), which, therefore, suggested a decrease in autophagy after ubenimex treatment.

To further verify the presence of autophagy in regulating HEXIM1 expression and JQ1 sensitivity, the cells were incubated with pharmacologic autophagy stimulator Rapamycin (10nm, together with ubenimex treatment). The data has shown a dramatically decreased HEXIM1 expression after upregulation of the autophagy level (Figure 5A). Immunofluorescence staining of U87M cells was performed to test JQ1 or JQ1 combined ubenimex treatment on HEXIM1 expression. The data showed that JQ1 treatment alone had no effect on HEXIM1 expression, however, in combination with ubenimex HEXIM1 expression was significantly down-regulated (Figure 5B, 5C). Min Huang et al. demonstrated that HEXIM1 is degraded through the process of autophagy. Our results are consistent with HEXIM1 autophagic degradation. The WST-1 assay verified that the effect of ubenimex-improved JQ1 sensitivity was attenuated (P<0.05, Figure 6A) upon knockdown of HEXIM1 in glioma cells. Moreover, the KD of HEXIM1 shifted the IC50 of JQ1 from 50nM to 125nM for U87M, from 235nM to 505nM for T98G and from 830nM to 4950nM for U251 (Figure 6B). All of the above data pointed to an important role of autophagy in ubenimex-regulated JQ1 sensitivity in glioma cells.

### The role of the Akt pathway in ubenimex-enhanced JQ1 sensitivity and invasion inhibition in glioma cells

#### The role of the Akt pathway in ubenimex-enhanced JQ1 sensitivity

The role of the Akt pathway in JQ1 sensitivity remains controversial. Cheng's study indicates higher Akt expression rescuing c-Myc from JQ1- induced suppression in glioblastoma, whereas tour his study indicates the opposite, c-Myc downregulation effects on Akt downregulation. Cheng's study also demonstrated that Akt-upregulation in the genetically engineered cells did not attenuate JQ1 sensitivity indicating that Akt may not directly associate with JQ1 resistance [19]. A pre-clinical analysis conducted by Vishal et al. suggested that JQ1 treatment could induce Akt suppression only at longer time intervals, indicating that the Akt pathway plays a context-depended role in JQ1 treatment of glioma cells [5].

**Figure 7:**
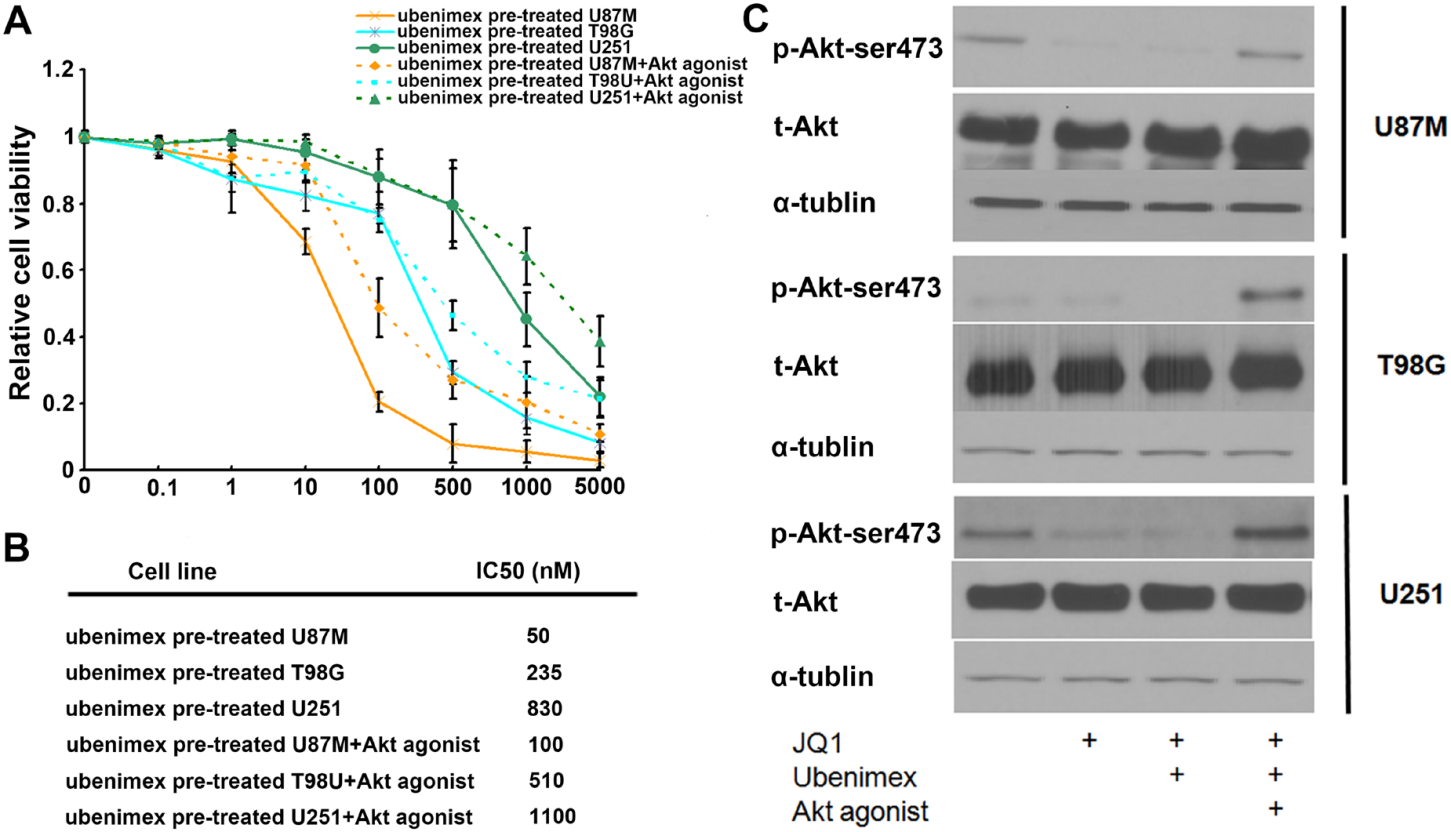
**(A)** WST-1 cell proliferation assay was used to test the effect of Akt on JQ1 sensitivity. **(B)** IC50 of JQ1 showed Akt stimulated by Akt agonist attenuated ubenimex enhanced JQ1 sensitivity. **(C)** The activity of Akt pathway was verified by using western blot. p-Akt-ser473 levels decreased by using ubenimex, but total Akt expression showed no difference.

Western blot showed that ubenimex treatment significantly reduced the concentration of phosphorylated proteins involved in the Akt signaling pathways (p-Akt-ser437), (Figure 7C). We designed a test to verify whether the Akt pathway is involved in JQ1 sensitivity in glioma cells. The WST-1 assay showed that treatment with an Akt agonist (10 nM) compromises JQ1 efficacy (P<0.01, Figure 7A). It also verified that ubenimex improved JQ1 sensitivity was attenuated after stimulating the Akt pathway using the Akt agonist (P<0.05, Figure 7A). The IC50 of JQ1 combined with the Akt agonist treatment shifted from 50nM to 100nM for U87M; from 235 nM to 510 nM for T98G and from 830nM to 1100nM for U251 (Figure 7B). The Akt agonist attenuated the ubenimex-enhanced JQ1 sensitivity suggesting that suppression of the Akt pathway by ubenimex is involved in JQ1sensitivity in glioma cells.

#### Combing JQ1 with ubenimex inhibit cell invasion in an Akt-dependent pathway

The Akt pathway plays an important role in regulating cell invasion. Our previous study verified that ubenimex could inhibit RCC cell migration by inhibiting the Akt pathway. Since one of the potential risks of JQ1 treatment is an aggressive progression of the tumor [5], a method to reduce glioma cell migration is essential in JQ1 treatment.

**Figure 8:**
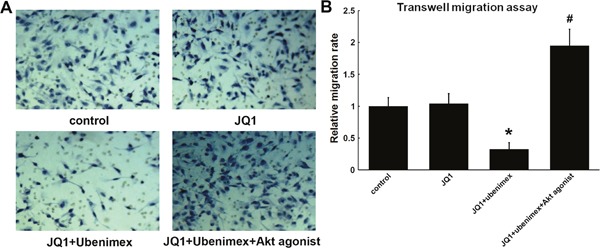
**(A)** Transwell migration assay showed JQ1 has no effect on migration of U251 glioma cells, however, combine with ubenimex treatment suppress migration of glioma cells. This effect can be attenuated by Akt agonist. **(B)** Quantitation of A (*P < 0.05 vs control, #: P<0.05 vs JQ1+ubenimex).

Trans-well assays were performed to determine whether ubenimex affects the migration capacity in glioma U251 cell lines. The migration capacity of glioma cells was significantly suppressed by combining ubenimex with JQ1 (P=0.012). The Akt agonist could significantly attenuate this effect (P=0.007), pointing to its role in suppressing glioma cell migration by combining ubenimex with JQ1 (Figure 8). However, the migration assay showed that there is no response to JQ1 treatment alone.

#### Ubenimex improved JQ1 efficiency in controlling the glioma tumor volume *in vivo*

**Figure 9:**
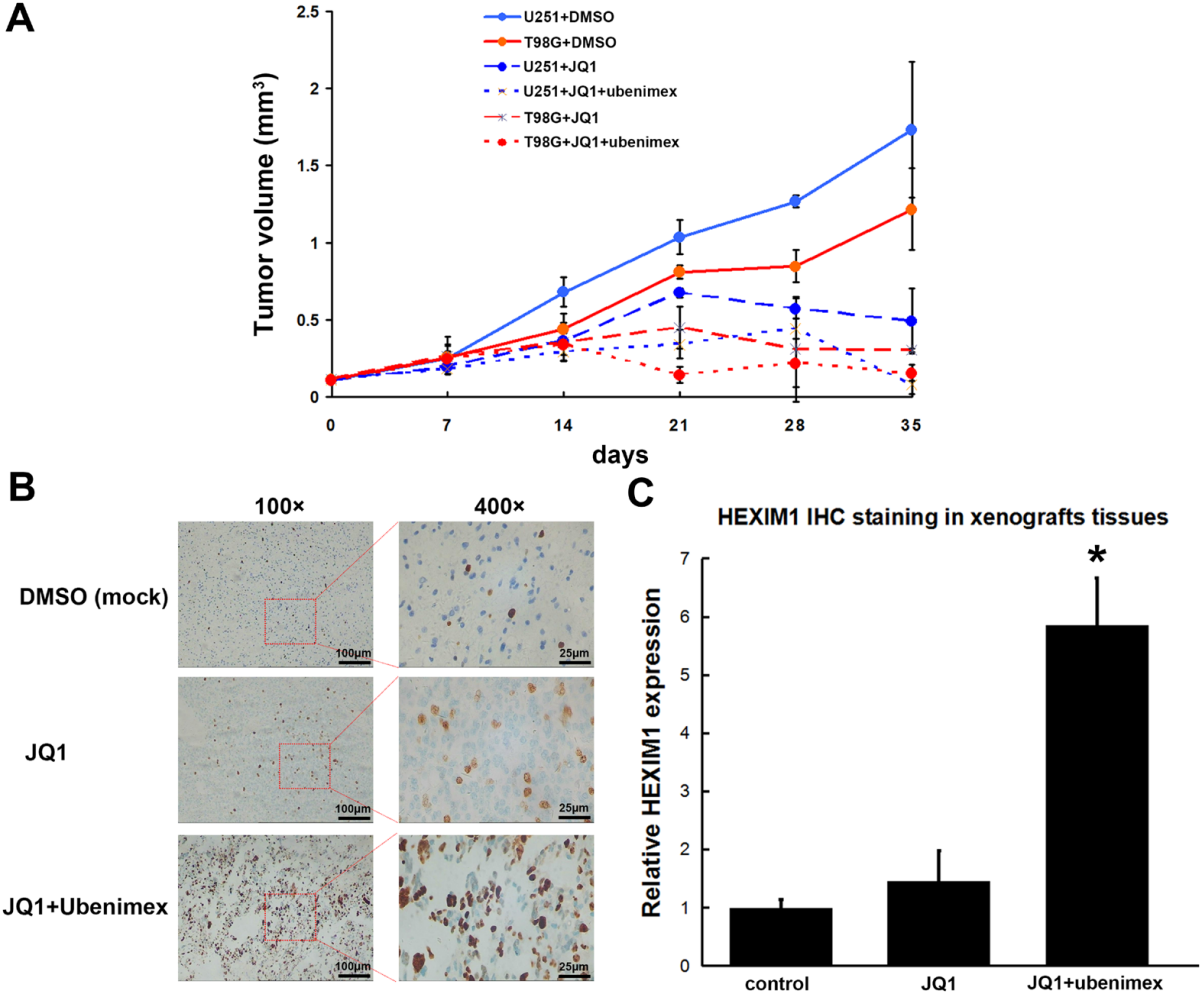
**(A)** Subcutaneous injection of 5×106 U251 or T98G cells per nude mouse was carried out. At 2 weeks (set as day 0), treatment was initiated and tumor lengths and widths were measured every 7 days. Tumor volume was derived as length × width 2/2. JQ1 at 30 mg/kg daily inhibited tumor growth; Combine with ubenimex significantly enhanced JQ1 efficiency. **(B)** HEXIM1 IHC staining was carried out, data showed HEXIM1 expression significantly improved after JQ1+Ubenimex treatment. **(C)** Quantitation of B (*P < 0.05 vs control).

*In vivo* experiments in mice showed that tumor volumes are controlled by JQ1 oral feeding. Two cell lines less sensitive to JQ1, T98G and U251, were tested using the xenograft model to check whether ubenimex could improve JQ1 efficiency *in vivo*. Consistent with the *in vitro* study results, T98G showed a better response to JQ1 treatment than the U251 xenografts model (P=0.017). In order to verify whether ubenimex could improve JQ1 sensitivity in less sensitive glioma cells, T98G, the U251 xenografts model was studied in more detail. The data showed that with ubenimex oral dosage, the volume of the tumor grew slower or shrunk much faster (P=0.031), indicating that ubenimex improved the efficiency of JQ1 in controlling the glioma tumor volume *in vivo* (Figure 9A). The tumor tissue from U251 xenografts was subjected to IHC staining to detect HEXIM1 expression. The data showed higher HEXIM1 expression in ubenimex-treated tumor tissues (P=0.015), which was consistent with *in vitro* studies (Figure 9B, 9C). In order to test whether ubenimex or JQ1 treatment could hinder the progression of glioma, dissection was performed after U251 xenografts were sacrificed. The metastases to lungs, the liver and bones were observed. The data showed that combined oral feeding with ubenimex significantly reduced the metastasis ratio compared to JQ1 treatment alone (0/20 vs. 3/20; P=0.002). However, no apparent difference between the JQ1 treatment group and the control group was observed (DMSO group, 4/20 vs. 3/20; P=0.003).

## DISCUSSION

Glioma, including glioblastoma (GBM), medulloblastoma, and oligodendrogliomas, typically originate in the brain or the spine. The potential of JQ1 in glioma has been reported during the past 10 years. Cheng found JQ1 induced marked G1 cell-cycle arrest and apoptosis in heterogeneous GBM as well as in orthotopic GBM tumors [19]. These studies suggest the therapeutic potential of JQ1 in GBM treatment. Henssen et al. evaluated the efficacy of JQ1 against medulloblastoma. They found that the JQ1 treatment significantly suppressed cell proliferation by decreasing cMYC activation [20]. Furthermore, Venkataraman's study demonstrated that BRD4 inhibition by JQ1 treatment in medulloblastoma induced apoptosis leading to a significant decrease in cell proliferation. However, the pre-clinical analysis showed its limitation by demonstrating that transient treatment with JQ1 leads to an aggressive development of the tumor and, therefore, accelerated death [5]. Thus, an improved understanding of the mechanisms underlying JQ1 is urgently required to design strategies to improve its efficiency, as well as overcome its limitations.

BRD4 regulates mitotic progression by binding to transcriptional start sites of genes and directing their post-mitotic transcription [21]. JQ1 gets incorporated in place of BRD4 fusion oncoprotein and inhibits its interaction with p-TEFb on chromatin, thus inducing cell-cycle arrest and initiating apoptosis. BRD4 mediates transcriptional elongation and has been linked to positive transcription elongation factor complex (P-TEFb)-induced increased growth promotion [22, 23].

**Figure 10:**
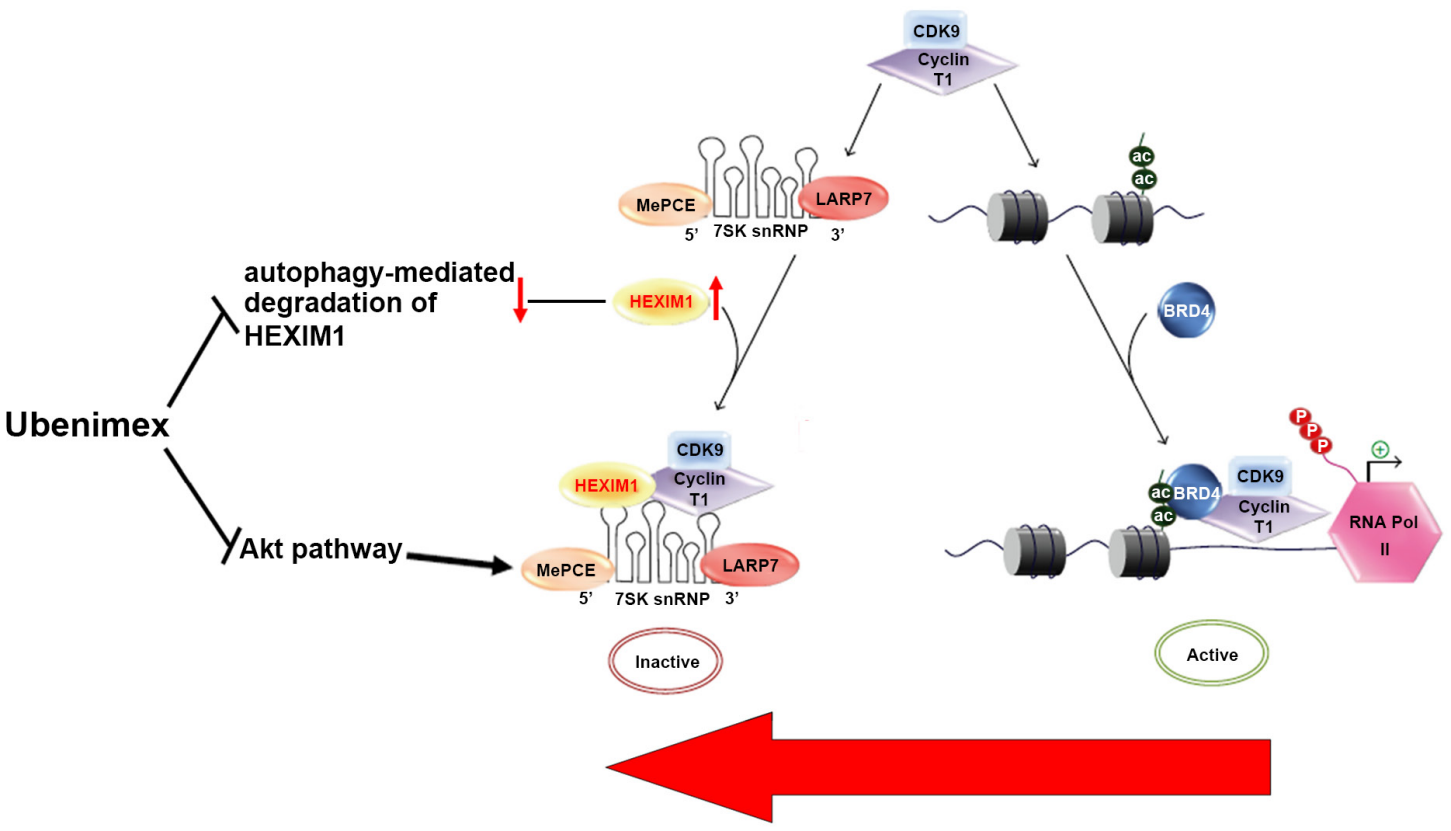
Proposed mechanisms by which ubenimex contributes to JQ1 sensitivity by pushing the equilibrium between the positive regulation of pTEFb by Brd4 and its negative regulation by HEXIM1 towards to pTEFb inactive via regulating autophagy and AKT →, positive regulation or activation; ┴, negative regulation or blockade.

Recent evidence demonstrated a role of HEXIM1 in cancers via regulation of P-TEFb dependent mechanisms [24, 25]. HEXIM1's major function is P-TEFb inhibition [26, 27]. It plays an equilibrium between the positive regulation by Brd4 and negative regulation by HEXIM1 of p-TEFb which determines the extent of BET activation [8, 9, 11, 12]. (Figure 10). Min Huang's research demonstrated a reduced sensitivity to JQ1 after degradation of HEXIM1 in a number of acute leukemia cell lines [14]. Our research confirmed that HEXIM1 is critical in regulating JQ1 sensitivity in glioma cells. HEXIM1 upregulation significantly improved JQ1 efficiency in inhibiting glioma cell lines’ proliferation and cell death.

Previous study showed ubenimex's benefits in glioma therapy [15], but its mechanism remains unclear. According to our previous study, ubenimex can inhibit the Akt pathway and regulate autophagy in renal cell carcinoma and prostate cells sensitive to radiotherapy [16–18]. We tested the effect of autophagy regulation and Akt inhibition in glioma cells. The data have shown that ubenimex can downregulate the level of autophagy and inhibit the Akt pathway. Besides, ubenimex showed significant potential in improving JQ1 sensitivity in glioma cells both *in vitro* and *in vivo*. Furthermore, we also tested whether this effect is associated with the autophagy suppression or Akt inhibition induced by ubenimex treatment.

Our study demonstrated that ubenimex treatment suppresses autophagy in glioma cells. Min Huang's research demonstrated the activation of autophagy as the cause of degradation of HEXIM1 in a subset of acute myelogenous leukemias, and verified its role in the regulation of HEXIM1 protein stability [14]. Autophagy activation and the subsequent proteolysis of HEXIM1 result in a shift in the balance between an inactive pTEFb-HEXIM1 complex and an active Brd4-pTEFb complex toward increased efficiency of Brd4 inhibition by JQ1. Our research show definitive evidence to suggest that autophagy is central to HEXIM1 degradation in a subset of glioma cells. The role of autophagy in the regulation of HEXIM1 protein expression was confirmed by a reversal of proteolysis, a decrease in LC3B expression, a decrease in autophagosome formation, and an increase in p62 - a key receptor of cargo uptake into the autophagosome vesicle.

The role of the Akt pathway in JQ1 sensitivity remains controversial. Haifeng Qiu's research demonstrated that JQ1 suppresses tumor growth via the PTEN/PI3K/AKT pathway in endometrial cancer. This research demonstrated that JQ1 blocks the PI3K/AKT pathway and suppress tumor growth. Thus, JQ1 resistance appears to be highly associated with the status of the Akt pathway in endometrial cancer [28]. However, Cheng demonstrated that Akt up-regulation does not attenuate JQ1 sensitivity, and therefore may suggest a lack of impact of Akt-activation on JQ1 resistance [19]. HEXIM1 was found to inhibit the pTEFb kinase activity by sequestering it in a complex with 7SK snRNA [7, 27], HMBA releases the P-TEFb from HEXIM1 and 7SK [13] (Figure 10), indicating a cross-talk between HEXIM1 and the Akt pathway. Our study showed that treatment with ubenimex significantly reduced phosphorylated proteins involved in the Akt signaling pathways (p-Akt-ser437), while treatment with an Akt agonist compromised JQ1 efficacy, indicating that the Akt pathway plays an important role in ubenimex-improved JQ1 sensitivity in glioma.

The Akt pathway also plays an important role in regulating the invasiveness of cancer cells. Our previous study demonstrated that ubenimex can suppress cell migration and invasion in prostate cancer and renal cell carcinoma cells via Akt inhibition. Vishal Rajagopalan showed that limitation of JQ1 treatment leads to aggressive tumor development and, therefore, accelerated death [5]. Our research demonstrated that JQ1 could suppress glioma cell's migration and invasion ability via downregulation of the Akt pathway, indicating that this adjuvant treatment could not only improve JQ1 efficiency but also overcome its limitation.

In conclusion, our results suggest the existence of a regulatory role of autophagy based degradation of HEXIM1 leading to BRD4 inhibition. Our study also provides a potential explanation for the BET inhibitors’ sensitivity and suggests that the adjuvant treatment with autophagy inhibitors such as ubenimex to JQ1 may be better than JQ1 treatment alone in glioma cells. Although the role of the Akt pathway in regulating JQ1 sensitivity remains controversial, our study demonstrated that ubenimex could improve JQ1 efficiency by suppressing the Akt pathway. Through suppression of the Akt pathway, the potential of ubenimex adjuvant treatment to attenuate parts of JQ1's side effects becomes evident. The potential of combining JQ1 with autophagy inhibition by ubenimex has also been confirmed in xenografts model, pointing to the utility of ubenimex adjuvant treatment in the treatment of glioma.

## MATERIALS AND METHODS

### Cancer cell lines and reagents

Glioma cells including U87M, T98G and U251 were purchased from ATCC (American type culture collection, USA) and cultured in RPMI 1640 medium with 5% fetal bovine serum (FBS) at 37°C in a humidified incubator containing 5% CO_2_. All the reagents used in this study were purchased from Sigma-Aldrich (St. Louis, MO), except for the enhanced chemiluminescence (ECL) detection reagents which were purchased from GE Healthcare (Piscataway, NJ), rabbit anti-pAkt (1:1,000, CST; Danvers, MA, USA), and rabbit anti-a tubulin (1:1,000, BOSTER, China)

### WST-1 cell proliferation assay

70% cells were seeded in 96-well plates at a density of 3,000 cells/well in 90 μl of complete medium supplemented with increasing concentrations of drugs. 72h after drug stimulation, 10 μl of WST-1 solution (WST-1 cell proliferation and cytotoxicity assay kit; Dojindo, Kumamoto, Japan) was added to each well. After one hour incubation at 37°C, absorbance at 450nm was determined using a microplate reader (EL340 Bio-Tek Instruments, Hopkinton, MA, USA).

The IC50 was measured by incubating the cells with a series of JQ1concentrations in 96-well plates according to the procedure similar to the WST-8 described below.

### Western blot

Parental and respective cells were seeded into 6-well plates and incubated with different concentrations of drugs. Before lysis, the cells were washed once with ice-cold PBS. Proteins were extracted from the cells with a suspension of RIPA buffer, 1% PMSF and 1% phosphatase inhibitor cocktail. Cells were scraped off, collected into eppendorf tubes and incubated on ice with a lysis buffer mixture for 30 minutes while shaking the mixture every 5 minutes. Extracted proteins were then centrifuged at 12,000rpm, and 4°C for 30 minutes and the supernatants were used for analysis. Protein concentrations were measured using the Bradford assay and the BCA protein assay kit (Solarbio, Beijing, China). Protein (40 μg) was electrophoresed on a pre-cast Bis-Tris polyacrylamide gel (8% and 12%) and then transferred to a PVDF membrane. The membranes were blotted with the following antibodies: rabbit anti-LC3B (1:1,000; Sigma), rabbit anti-HEXIM1 (1:1,000, sigma, USA), rabbit anti-Akt (1:1,000, CST; Danvers, MA, USA), rabbit anti-pAkt (1:1,000, CST; Danvers, MA, USA), and rabbit anti-a tubulin (1:1,000, BOSTER, China), followed by horseradish peroxidase (HRP)-conjugated secondary antibodies (1:5,000; ZB2306 and ZB2301; both from ZsBio, Beijing, China). Immunoblots were developed by enhanced chemiluminescence (LAS4000).

### Immunocytochemistry

The coverslips containing fixed cells were washed two times with wash buffer. Non-specific staining was blocked by adding blocking buffer and incubating the cells for 45 minutes at room temperature. Following, the blocking buffer was removed and the primary antibody rabbit anti-HEXIM1 (1:300, sigma, USA) was diluted in dilution buffer. The cells were then incubated overnight at 4°C. The secondary antibody was diluted in dilution buffer and 400 μL were added to the wells and incubated at room temperature for 1 hour in the dark. One drop of the anti-fade mounting medium was dispensed onto the microscope slide per coverslip. The coverslip was mounted with the cells facing towards the microscope slide and visualized using a fluorescence microscope (OLYMPUS, Japan).

### Annexin V-fluorescein isothiocyanate (FITC)/propidium iodide (PI) staining

Apoptotic cells were quantified (%) using an annexin V-FITC/PI kit (Nanjing KeyGen Biotech, Co. Ltd., Nanjing, China) and detected by flow cytometry. The cells were harvested after 12h of treatment with JQ1 alone or in combination with ubenimex. Next, the cells were resuspended in the binding buffer (10 mmol/l 4-(2-hydroxyethyl)-1-piperazineethanesulfonic acid, 140 mmol/l NaCl and 2.5 mmol/l CaCl2, pH 7.4) and incubated with annexin V-FITC/PI in the dark for 15 min. A total of 5,000 cells/sample were analyzed using an FACSCalibur or an EPICS XL flow cytometer (BD Biosciences, Franklin Lakes, NJ, USA). The cells in the early stages of apoptosis stained positive for annexin V-FITC, whereas those in the late stages of apoptosis stained positive for both annexin V-FITC and PI.

### Acridine orange (AO)/ethidium bromide (EB) double staining

Cells were cultured in 6-well plates for 24 h and were then treated with different doses of ubenimex for 16 h. After the indicated treatment times, AO/EB operating fluid was mixed reagent A, reagent B, and reagent C at a certain rate of 1:1:8. Each sample was added 2ul AO/EB operating fluid, discarded the supernatant after low speed centrifugal (500g/min), re-suspend cells in AO/EB Dilution Buffer at a concentration of 5×10^6 cells/ml, then add 1ul AO/EB operating fluid to 25ul cell suspension. Next, the cells were observed under a fluorescence microscope (Nikon, Inc., Japan).

### Electronic microscopy

After incubation, the cells were fixed with 2.5% glutaraldehyde for 1.5h, postfixed with 1% osmium tetroxide for 1.5h, dehydrated with increasing ethanol, embedded and sectioned. Sections were stained with Uranium Acetate and 0.3% lead citrate and observed on a JEOL-1200EX electron microscope (Jeol, Tokyo, Japan).

### Animal experiments

Five weeks old female nude BALB/c mice were obtained from VITAL RIVER (Beijing, China). The study protocol was approved by the Animal Ethics Committee of the Provincial Hospital Affiliated to Shandong University and in line with the guidelines of the Office of Laboratory Animal Welfare. The experiment schedule was designed on the basis of preliminary experiments. Approximately 5×106 ACHN/ACHN-R cells were subcutaneously inoculated into the right flank of mice which was randomly arranged into five groups of five mice per group. Two weeks later, the mice were treated on day 0 once daily by gavage with either vehicle (DMSO), JQ1 (30mg/kg), JQ1+ ubenimex (20mg/kg) in both parental and resistance groups. The tumor volumes were estimated every 7 days using the standard formula: volume = length × width 2/2. One month later, all mice were sacrificed.

### Immunohistochemical staining (IHC)

Tumor tissues from mice were formalin-fixed and paraffin-embedded. After rehydration and antigen retrieval, the slides (thickness = 4 μm) were incubated with primary antibodies: anti-HEXIM1 (1:50). The staining was visualized using DAB (Invitrogen, CA) and each slide was scored by two pathologists.

### Statistical analysis

Student's t-test was used for statistical analysis of data. The analysis was performed using the Statistical Package for Social Science (SPSS for Windows package release 10.0; SPSS Inc., Chicago, IL, USA). P<0.05 was considered statistically significant.
